# Synchronous profiling and analysis of mRNAs and ncRNAs in the dermal papilla cells from cashmere goats

**DOI:** 10.1186/s12864-019-5861-4

**Published:** 2019-06-20

**Authors:** Sen Ma, Ying Wang, Guangxian Zhou, Yi Ding, Yuxin Yang, Xiaolong Wang, Enping Zhang, Yulin Chen

**Affiliations:** 1 College of Animal Science and Technology, Northwest A&F University, Yangling, 712100 Shaanxi China; 2 Department of Animal Science, Guangdong Ocean University, Zhanjiang, 524088 Guangdong China

**Keywords:** NcRNAs, Dermal papilla cells, Cashmere goats, Hair follicle, *HOXC8*, *RSPO1*, ceRNAs, Hair follicle stem cells

## Abstract

**Background:**

Dermal papilla cells (DPCs), the “signaling center” of hair follicle (HF), delicately master continual growth of hair in mammals including cashmere, the fine fiber annually produced by secondary HF embedded in cashmere goat skins. Such unparalleled capacity bases on their exquisite character in instructing the cellular activity of hair-forming keratinocytes via secreting numerous molecular signals. Past studies suggested microRNA (miRNAs) and long non-coding RNAs (lncRNAs) play essential roles in a wide variety of biological process, including HF cycling. However, their roles and related molecular mechanisms in modulating DPCs secretory activities are still poorly understood.

**Results:**

Here, we separately cultivated DPCs and their functionally and morphologically distinct dermal fibroblasts (DFs) from cashmere goat skins at anagen. With the advantage of high throughput RNA-seq, we synchronously identified 2540 lncRNAs and 536 miRNAs from two types of cellular samples at 4th passages. Compared with DFs, 1286 mRNAs, 18 lncRNAs, and 42 miRNAs were upregulated, while 1254 mRNAs, 53 lncRNAs and 44 miRNAs were downregulated in DPCs. Through overlapping with mice data, we ultimately defined 25 core signatures of DPCs, including *HOXC8* and *RSPO1*, two crucial activators for hair follicle stem cells (HFSCs). Subsequently, we emphatically investigated the impacts of miRNAs and lncRNAs (*cis*- and *trans*- acting) on the genes, indicating that ncRNAs extensively exert negative and positive effects on their expressions. Furthermore, we screened lncRNAs acting as competing endogenous RNAs (ceRNAs) to sponge miRNAs and relief their repressive effects on targeted genes, and constructed related lncRNAs-miRNAs-*HOXC8*/*RSPO1* interactive lines using bioinformatic tools. As a result, XR_310320.3-chi-miR-144-5p-*HOXC8*, XR_311077.2-novel_624-*RSPO1* and others lines appeared, displaying that lncRNAs might serve as ceRNAs to indirectly adjust HFSCs status in hair growth.

**Conclusion:**

The present study provides an unprecedented inventory of lncRNAs, miRNAs and mRNAs in goat DPCs and DFs. We also exhibit some miRNAs and lncRNAs potentially participate in the modulation of HFSCs activation via delicately adjusting core signatures of DPCs. Our report shines new light on the latent roles and underlying molecular mechanisms of ncRNAs on hair growth.

**Electronic supplementary material:**

The online version of this article (10.1186/s12864-019-5861-4) contains supplementary material, which is available to authorized users.

## Background

Cashmere goats gain worldwide reputation for yielding cashmere, the fine hair fiber with excellent quality and commercial value, from the secondary hair follicle (HF) of skins [1–3]. An outstanding feature of cashmere growth is annual rhythm, which synchronously occurs with yearly sequential switches of HF among fast growth stage (anagen), gradual degeneration stage (catagen), and relative quiescence stage (telogen) [4–6]. Although cashmere elongation tightly synchronizes with the alterations of photoperiod and endocrine status, the basic rationales are similar with other mammals [7]. Cyclic transformations of follicular keratinocytes among proliferation, differentiation and apoptosis are the cellular foundations of periodical HF regrowth [8]. However, the specialized fibroblasts locating at the bottom of HF, dermal papilla cells (DPCs), are widely accepted as the controlling center of HF growth. Via spatiotemporally releasing specific signals,

DPCs can finely instruct the activities of follicular keratinocytes to reshape HF and produce new hair shaft. DPCs also serve as a transfer station to relay the effects of locally or systematically generated hormones and other molecules on hair growth [9]. Past studies suggested that the particular ability of DPCs on HF cycling resides in their characteristic gene expression profiles, and identified some involved key regulators and signaling pathways such as *Sox2*, *Prdm1*, *β-catenin*, Wnt/β-catenin pathway, FGF pathways and others [10–14]. Whereas, the current picture of overall landscape is far from complete, especially for the versatile regulatory non-coding RNAs (ncRNAs).

Long thought as “evolutionary junk”, nowadays ncRNAs have been continually implicated to play irreplaceably regulatory roles in gene expression [15]. In contrast to functionally monotonous, microRNAs (miRNAs), long non-coding RNAs (lncRNAs), which are typically defined as RNA transcripts > 200 nucleotides in length, can actively modulate gene expression through various mechanisms that are not yet explicitly understood [16]. LncRNAs extensively exert their regulatory roles at transcriptional and posttranscriptional levels and play indispensable roles in a number of vital developmental and pathological processes, such as cell differentiation [17], organogenesis [18], X-chromosome imprinting [19], stem cells pluripotent maintenance [20], and carcinogenesis [21]. Recently, a mounting quantity of research has revealed that ncRNAs are the essential participators of HF development and functionality maintenance of DPCs. Epidermal specific deletion of Dicer, the necessary miRNAs-processing enzyme, resulted in stunned HF formation and hyperproliferative follicular cells [22]. Inducible overexpression of miR-214 drastically inhibited HF development and cycling via specifically decreasing the expression of β-catenin, the key Wnt signaling mediator [23]. Moreover, alteration of miRNAs expression profiles were frequently associated with the loss of hair-modulatory functions of DPCs on humans and mice [24, 25]. Some miRNAs such as miRNA-125b and miRNA-195-5p have been confirmed as the casual factors through restraining growth factors expressions or weakening intensity of Wnt pathway, the primary pivotal signaling inside DPCs [26, 27]. LncRNAs also make a considerable contribution to ncRNAs-mediated manipulation of hair cycling, though less explored. Several reports revealed that the expression of lncRNAs experiences obvious fluctuations during stage transitions of cashmere cyclic growth [2, 5]. A few lncRNAs such as H19, HOTAIR and lncRNA-000133 have been shown to regulate genes closely involved in cyclic HF growth in DPCs [28, 29]. Apart from functioning individually, lncRNAs can serve as competing endogenous RNAs (ceRNAs) to specifically decoy miRNAs for preventing their suppression on targeted genes [30, 31]. Such feature of lncRNAs facilitates more precise and complex regulations of gene expression, which have been uncovered to relate with a series of physiological events and cancer progressions [32–34], and might contribute to the exquisite modulation of DPCs functionality.

Though a few studies suggest ncRNAs are important regulators of hair cycling on cashmere goats, they mainly focused on skin tissue, a complex structure comprising a dozen of cell types [3, 5, 35]. There are still no reports on the expression profiles and the potential roles of ncRNAs in goat DPCs. Otherwise, previous researchers rarely executed simultaneous mRNAs, miRNAs and lncRNAs analysis from identical DPCs sample sets either on mice or other animals, leading to insufficient interpretation of lncRNAs performing as the newly proposed ceRNAs. In the present trial, we concurrently profiled above transcripts from DPCs and dermal fibroblasts (DFs) cultivated from anagen cashmere goat skins. Using bioinformatic tools, we subsequently screened key genes and ncRNAs, and constructed their interactive networks to highlight the presumed functions of ncRNAs in hair growth, especially for hair follicle stem cells (HFSCs) activation. Our study will provide novel clues and viewpoints to deeply explore the unrecognized functions of ncRNAs in DPCs-centered hair growth.

## Results

### Cultivation and morphological characterization of goat DPCs and DFs

**Fig. 1:**
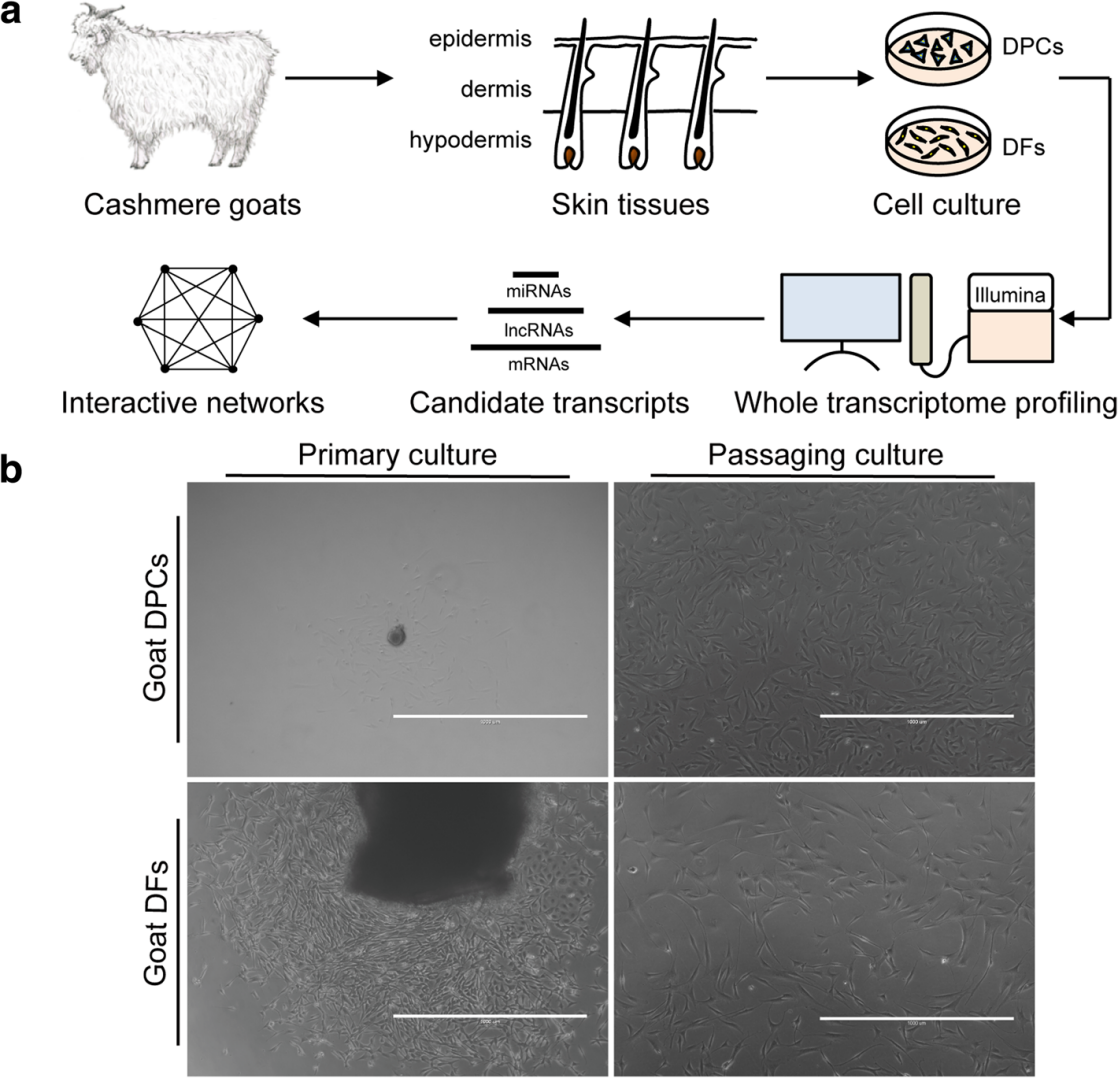
Schematic presentation of the overall workflow in the present study (**a**) and in vitro cultivation of DPCs and DFs (**b**). Cells migrated out from each explant in 5–7 days, and their subcultures showed remarkably different morphologies: DPCs are relatively flat, whereas DFs show a spindle-like cellular shape. Scale bar = 1000 μm

In vitro cultured DPCs specifically supported HF neogenesis and hair regrowth, whereas their relatives DFs did not [36]. To comprehensively gain insights into the innate feature of this specialized cell type, we designed the present study to culture and molecularly characterize both cell populations from lateral backsides skins of 2-year-old female cashmere goats at anagen (Fig. 1a). Both populations outgrew from their respective explant in 5–7 days, and their passaging cultures obviously exhibited distinct cellular morphologies (Fig. 1b). DPCs are flat in appearance, with spread-out surfaces and multiple cellular projections, whereas DFs typically show a bipolar and spindle-like pattern, possessing a relatively smaller cell volume. These morphological features in goats are consistent with mice and other mammals [37, 38], thereby implying the successful cultivations of related cells and the high fidelity of subsequent analysis.

### Comprehensive identification of ncRNAs in goat DPCs and DFs

As previously reported, when cultured in vitro goat DPCs at early passages formed a sphere-like aggregate, an indicator of their hair-inducing capacities, whereas DFs did not possess such ability [37, 39, 40]. To uncover the ncRNAs and mRNAs underlying the morphological and functional differences between DPCs and DFs, we selected both cell types at 4th passages and synchronously sequenced six cell samples (*n* = 3 for each type) on Illumina HiSeq 4000 (for mRNAs and lncRNAs) and HiSeq 2000 platforms (for miRNAs). Standard bioinformatic analysis procedures were carried out as previous studies [2, 5].

As a result, we obtained an average of 114,777,434 and 119,977,164,150 bp paired-end raw reads for each DPCs and DFs sample, respectively. When invalid reads were intentionally excluded as usual, filtered clean reads (accounting for 98.5% of raw reads) were mapped to the goat reference genome [41]. After the assembly and quantification of the linear transcripts using StringTie (v1.3.1) [42] and Cuffdiff (v2.1.1) [43]**,** respectively, we distinguished mRNAs and lncRNAs through five successive sifting steps. The detailed procedures and results are shown in Additional file 1: Figure S1a). In the final step, three tools CPC [44], Pfam [45] and CNCI [46], were jointly applied to fully eliminate transcripts with uncertain coding potential, ensuring the credible discovery of fresh lncRNAs (Additional file 1: Figure S1b). Finally, 786 annotated lncRNAs and 1754 novel ones were recognized in all samples. A total of 22,972 mRNAs were also discerned.

As for miRNAs profiling, an average of 14,555,565 and 12,621,142 50 bp single-end raw reads were acquired from each DPCs and DFs sample, respectively. Filtered as reported before [2], clean reads (accounting for 97.73% of raw reads) with certain ranges of length (18–35 nt) were mapped to reference sequences [41], and mapped tags were searched within miRBase to sort out known miRNAs. In total, 397 mature known miRNAs were found. We further used two available software programs miREvo [47] and mirdeep2 [48], to speculate novel miRNAs based on a featured hairpin structure, the Dicer cleavage site, etc.; ultimately, 139 novel transcripts were predicted. Collectively, we discovered 536 expressed mature miRNAs in all samples.

Numbers of mRNAs and ncRNAs are summarized in Table 1. Genomic information of lncRNAs is detailed in Additional file 2: Table S1.

**Table 1:** Information of known and novel transcripts in DPCs and DFs samples

Item	mRNAs Transcript/Gene	LncRNAs Transcript/Gene	Mature miRNAs
Known	22,972/12071	786/639	397
Novel	–	1754/1466	139
Total	22,972/12071	2540/2105	536

“-” represents transcripts were not available in annotation files

### Genomic feature comparison between lncRNAs and mRNAs

To ascertain the results of transcripts identification and supplement the annotation information of goat genome, we compared several noticeable genomic features between lncRNAs and mRNAs. The analytical results indicated that annotated lncRNAs normally contain more exons than novel lncRNAs, although fewer than mRNAs (Additional file 3: Figure S2a). Lengths of these transcripts showed an obviously distinct pattern, in which annotated and novel lncRNAs are shorter and more narrowly distributed than mRNAs (Additional file 3: Figure S2b). Moreover, the overall expression levels of both lncRNAs were significantly lower than mRNAs, and the newly identified lncRNAs were the lowest (Additional file 3: Figure S2c). These genomic traits are highly in accordance with previous reports on goats and other species [49–52], suggesting the credible profiling of lncRNAs in present trial.

### Differentially expressed mRNAs and ncRNAs

**Fig. 2:**
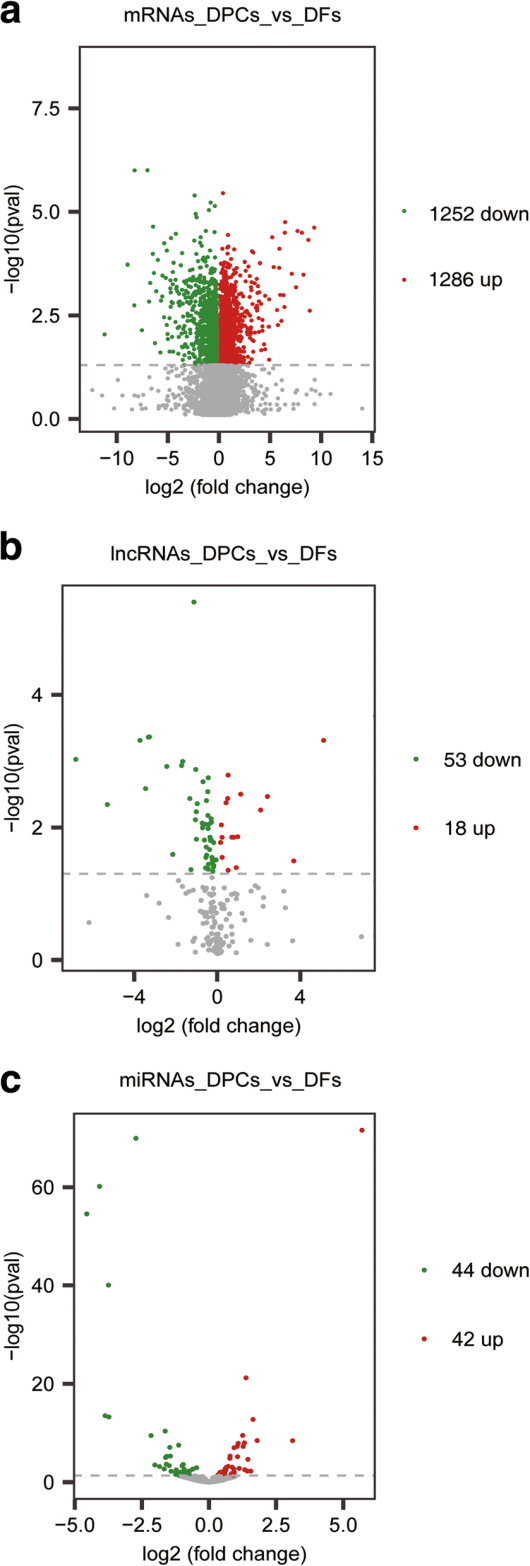
Differentially expressed ncRNAs and mRNAs between goat DPCs and DFs. **a** Differentially expressed mRNAs. **b** Differentially expressed lncRNAs. **c** Differentially expressed miRNAs. Among these mRNAs and ncRNAs, 1286 mRNAs, 17 lncRNAs, 42 miRNAs were upregulated, and 1254 mRNAs, 53 lncRNAs and 44 miRNAs were downregulated in DPCs compared with DFs. Red dots represent upregulated transcripts and green dots represent downregulated transcripts in DPCs compared with DFs

To fulfill the purpose for selecting potential candidates related to hair growth, differentially expressed mRNAs and ncRNAs were examined between two sets of samples using Ballgown suite [53]**.** As visualized by heatmaps in Additional file 4: Figure S3, three samples perfectly gathered inside DPCs and DFs groups. Volcano plots in Fig. 2 graphically displayed fold changes and statistical significances of entire transcripts. In total, 1286 up-regulated and 1252 down-regulated mRNAs emerged in goat DPCs compared with DFs. Then, we checked the expressions of several genes frequently utilized for in vivo labelling of DPCs on mice, and discovered *HOXB6*, *ENPP2*, *CRABP1* and *PRDM1* are highly expressed in goat DPCs. Among them, *CRABP1* is a constant marker of DPCs, and expresses throughout entire stages of hair cycling [54]. Of note, *COL15A1*, one of top expressed genes, is solely abundant in DPCs. Such condition is in consistent with the discovery that DFs do not produce COL15A1 protein in culture dishes [55]. Most importantly, we defined the core signatures of DPCs through overlapping with mice data [56], and as a result a total of 25 gene emerged (Table 2). The reason for a relatively fewer number of genes is that the mice markers were screened through comparing with other four cell lineages including melanocytes, outer root sheath cells, HMCs and DFs. The gene list contains *LEPR*, *WNT5A*, *PRDM1*, *HOXC8*, *FGFR2, BMP4, LTBP1,* and *PTGFR.* They almost participate in all pivotal aspects of DPCs functions in HF cycling, such as HFSCs activation, hair matrix cells (HMCs) proliferation and differentiation, angiogenesis, and hormone-mediated hair growth regulation. Moreover, we also detected that two hormone receptors *PTGER4* and *ESR1* are more abundant in DPCs, as reported before [57, 58].

**Table 2:** Information of 25 core signatures in goat DPCs and DFs

Gene name	Gene_id	Transcript_id	DPCs_FPKM	DFs_FPKM	log_2_(foldchange)	*p* value	qvalue
COL15A1	100,860,925	XM_018052232.1	11.04568	0	inf	0.003718	0.051566
SPP1	100,860,805	XM_013964504.2	63.68734	4.234169	3.910856	0.001439	0.038888
LEPR	102,180,987	XM_018045220.1	35.23822	2.352076	3.905134	0.007887	0.068539
SPP1	100,860,805	NM_001285667.1	73.68631	5.486852	3.747346	0.002121	0.043653
WNT5A	102,170,921	XM_018038230.1	2.88584	0.408166	2.821762	0.007041	0.066033
WNT5A	102,170,921	XM_018038229.1	3.770505	0.639787	2.559094	0.005662	0.06086
LTBP1	102,190,794	XM_018055104.1	5.145034	0.967109	2.41143	0.032229	0.142671
WNT5A	102,170,921	XM_018038231.1	76.81172	15.48829	2.310149	0.004212	0.054011
CRABP1	102,182,914	XM_005695096.3	2.244416	0.489605	2.196649	0.046864	0.174763
FGFR2	102,170,938	XM_018041429.1	2.262661	0.518313	2.126126	0.02273	0.117895
PTGFR	102,173,625	XM_005678194.3	14.90073	3.503655	2.088451	0.013132	0.087373
LEPR	102,180,987	XM_018045222.1	5.766132	1.364422	2.079314	0.029657	0.135528
PRDM1	102,181,213	XM_018053115.1	2.454423	0.624763	1.974003	0.012663	0.085148
PDGFRA	102,174,007	XM_013964633.2	18.06262	5.869759	1.621634	0.00426	0.054182
INHBA	100,860,960	XM_013963334.2	6.395766	2.093363	1.611294	0.031367	0.140456
PDGFRA	102,174,007	XM_005681627.3	326.9543	142.4327	1.198809	0.000685	0.033058
S100B	102,169,524	XM_005675677.3	71.57221	32.04991	1.159079	0.013184	0.087499
PTK7	102,173,428	XM_018038593.1	61.50198	28.91053	1.089038	0.000717	0.033241
TMEM100	102,177,089	XM_018064140.1	6.607991	3.262818	1.018093	0.036158	0.150997
STEAP1	102,177,954	XM_005679304.3	90.92992	48.45148	0.908214	0.008387	0.070022
PAPPA	102,179,947	XM_018052683.1	6.921659	3.830993	0.853399	0.020293	0.111103
ENPP2	102,176,626	XM_018058157.1	14.34361	7.985599	0.844935	0.03353	0.145215
RSPO1	100,860,770	XM_018039333.1	18.48713	10.33948	0.838357	0.022258	0.11657
TWIST1	102,182,849	XM_018047345.1	25.40524	15.11656	0.748996	0.001425	0.038888
HOXC8	102,177,136	XM_005680080.3	10.12046	6.152629	0.718	0.006587	0.064298
BMP4	100,860,789	XM_013967192.2	10.11324	6.192798	0.707582	0.006081	0.062505
BMP4	100,860,789	NM_001285646.1	42.12438	27.82585	0.59823	0.004085	0.05347
DRAM1	102,187,197	XM_005680638.3	84.57294	58.46341	0.532662	0.000442	0.030155
SCARA3	102,181,778	XM_018051874.1	11.80534	8.397271	0.491447	0.023285	0.119601
MAGED2	100,860,788	XM_013976557.2	11.89109	8.886352	0.420218	0.022344	0.116912
GNL2	102,174,356	XM_005678681.3	49.33153	39.51102	0.320255	0.007745	0.067889
MAGED2	100,860,788	NM_001285645.1	112.3633	90.58544	0.310819	0.014187	0.090599

A relatively less quantity of 71 lncRNAs exhibited varied abundances between two different functional cell populations. Of 18 elevated lncRNAs in DPCs, XR_001296062.1, LNC_001710, XR_001917771.1 and LNC_000335 showed exclusive expressions. Likewise, LNC_000726, LNC_000799, XR_310887.3, LNC_000964, LNC_000901, and LNC_000299 are uniquely occupied by DFs among the remaining 53 downregulated lncRNAs. These results indicated that lncRNAs may regulate DPCs functionality and further hair cycling via their cell-type restricted presences.

As for miRNAs analysis, we found 86 miRNAs have significantly different abundances between goat DPCs and DFs. Among them, 42 and 44 miRNAs showed upregulated and downregulated expressions in DPCs compared with DFs, respectively. Some miRNAs, such as miR-125b [24] and miR-196a [26] have been documented with regulatory roles in DPCs functions on human and mice.

We listed top 20 (10 upregulated and 10 downregulated) differently expressed lncRNAs and miRNAs in Table 3. Holistic lists of mRNAs and ncRNAs and more details are provided in Additional file 5: Table S2.

**Table 3:** Top 20 differentially expressed ncRNAs in goat DPCs and DFs

Transcript_id	gene_id	DPCs_FPKM/readcount	DFs_FPKM/readcount	log_2_(foldchange)	*p* value	qvalue
lncRNAs						
XR_001296062.1	1.02E+ 08	1.99008	0	inf	0.000207	0.025161
LNC_001710	XLOC_075943	5.239176	0.150354	5.122901	0.000488	0.030155
XR_001917771.1	1.02E+ 08	1.772177	0.137917	3.68365	0.032047	0.142154
LNC_000335	XLOC_013190	2.363259	0.441866	2.419098	0.003407	0.050636
XR_001295493.2	1.02E+ 08	7.632797	1.791479	2.091061	0.005455	0.060082
XR_001295577.2	1.02E+ 08	12.35869	5.636179	1.132737	0.003141	0.049678
XR_001918397.1	1.09E+ 08	13.92489	7.030079	0.986053	0.013808	0.089779
XR_001917946.1	1.02E+ 08	3.059054	1.620502	0.916644	0.040454	0.160805
XR_001297370.2	1.07E+ 08	23.97548	13.86575	0.790034	0.014074	0.090362
XR_001917801.1	1.09E+ 08	7.39471	4.537434	0.704617	0.014055	0.090362
LNC_000299	XLOC_011942	0.572715	2.523932	−2.13978	0.025557	0.125133
XR_001296495.2	1.02E+ 08	1.594773	8.587425	−2.42887	0.001211	0.036929
LNC_000901	XLOC_040539	0.265664	2.556129	−3.26628	0.000433	0.030155
XR_001918151.1	1.09E+ 08	1.081655	10.75737	−3.31401	0.000437	0.030155
LNC_000726	XLOC_032789	0.220355	2.407738	−3.44978	0.002588	0.046272
LNC_000799	XLOC_035825	0.95753	12.62068	−3.72033	0.000488	0.030155
XR_310887.3	1.02E+ 08	0.105149	4.113782	−5.28996	0.004512	0.055433
LNC_000964	XLOC_044020	0.024105	2.700893	−6.80798	0.000945	0.034269
LNC_000474	XLOC_019528	0	1.968306		0.002211	0.043714
LNC_000834	XLOC_037289	0	2.67851		0.004903	0.057038
miRNAs						
chi-miR-196b		491.8586	6.14035	5.6981	2.89E-72	1.08E-69
novel_144		20.20519	0.34253	3.1137	3.87E-09	1.03E-07
chi-miR-504		163.8782	42.25665	1.7943	3.72E-09	1.03E-07
chi-miR-10a-5p		74,479.94	22,786.8	1.6405	1.77E-13	7.37E-12
chi-let-7 g-3p		4.720801	0.367675	1.5577	0.006164	0.046103
chi-miR-2483-5p		42.33788	13.62568	1.4483	2.48E-05	0.000332
chi-miR-2483-3p		9.504497	2.10547	1.4247	0.005922	0.045198
chi-miR-450-5p		1632.57	617.3818	1.3789	6.09E-22	3.79E-20
chi-miR-449b-3p		24.99155	7.64879	1.3302	0.003109	0.025835
chi-miR-424-3p		240.5518	91.92476	1.3181	1.07E-08	2.68E-07
novel_549		1.124597	7.295191	−1.671	0.002183	0.01991
chi-miR-497-3p		1.025707	8.638641	−1.8433	0.000716	0.007648
chi-miR-206		0.526303	9.17283	−2.0183	0.000352	0.004241
chi-miR-497-5p		20.19273	108.0983	−2.1601	3.50E-10	1.09E-08
chi-miR-9-5p		788.4207	5396.696	−2.7232	1.11E-70	2.08E-68
chi-miR-99a-3p		1.025707	48.28177	−3.7294	5.73E-14	2.68E-12
chi-miR-125b-3p		47.34329	760.8219	−3.7448	9.08E-41	6.79E-39
chi-let-7c-3p		0.802904	53.93692	−3.8687	3.20E-14	1.71E-12
chi-miR-9-3p		20.93235	416.4388	−4.0787	7.04E-61	8.78E-59
chi-miR-99a-5p		1572.609	46,948.92	−4.5546	2.87E-55	2.68E-53

To assure the accuracy of sequencing strategy, bioinformatic identifications and analysis, we randomly picked several differentially expressed mRNAs (*ESR1*, *INHBA*, *INHBB*, *IGFBP2*, *LEPR*, *BMP4*, *THBS1*, *WNT2*, and *WNT5A*) and lncRNAs (*LOC102172689*, *LOC106503672*, *JAM3*, and *LOC102171315*), and used qPCR to ascertain their relative levels between the two sample groups. The expression levels of transcripts determined by qPCR and RNA-seq are highly consistent (Additional file 6: Figure S4), thereby implying the prominent reliability of RNA-seq data acquisitions and following analytical procedures in present study.

### KEGG pathway enrichment analysis

To better understand the functioning approaches of DPCs in hair growth, KEGG analysis for upregulated mRNAs is performed, and finally 247 pathways were enriched. Of the 20 top pathways, focal adhesion and ECM-receptor interaction frequently appeared in transcriptomic studies on goat skin tissues and DPCs on mice [3, 59, 60]. Most importantly, several hormone-related pathways (Estrogen signaling pathway, Thyroid hormone signaling and Adipocytokine signaling pathway) draw our special attention, though they are not significantly enriched. There are two reasons: one is all of them were emphasized during stage transitions of cashmere growth in skins by researchers before [2, 5]; the other is corresponding hormone-binding receptors (i.e., *ESR1*, *IGTAV* and *LEPR*) were on the list of upregulated genes, which strongly suggests goat DPCs are targets of estrogen, thyroid hormone, and leptin in the HF. Full list of pathways and their involved genes are shown in Additional file 7: Table S3.

### Prediction of lncRNAs and miRNAs on the regulation of core signatures

**Fig. 3:**
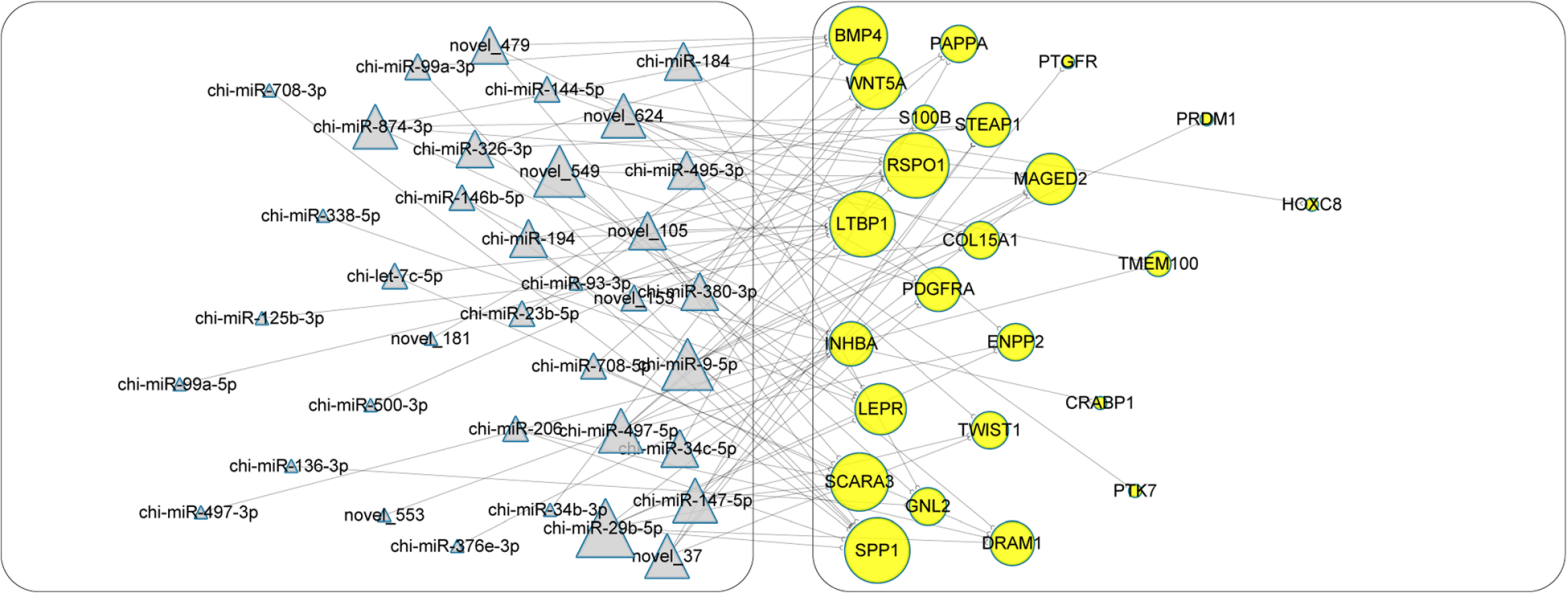
Interactive map of miRNAs with core signatures. Triangles with grey color indicate downregulated miRNAs in DPCs compared with DFs, and circles with yellow color indicate core signature of goat DPCs. The size of each shape positively correlates with the number of connected lines

**Fig. 4:**
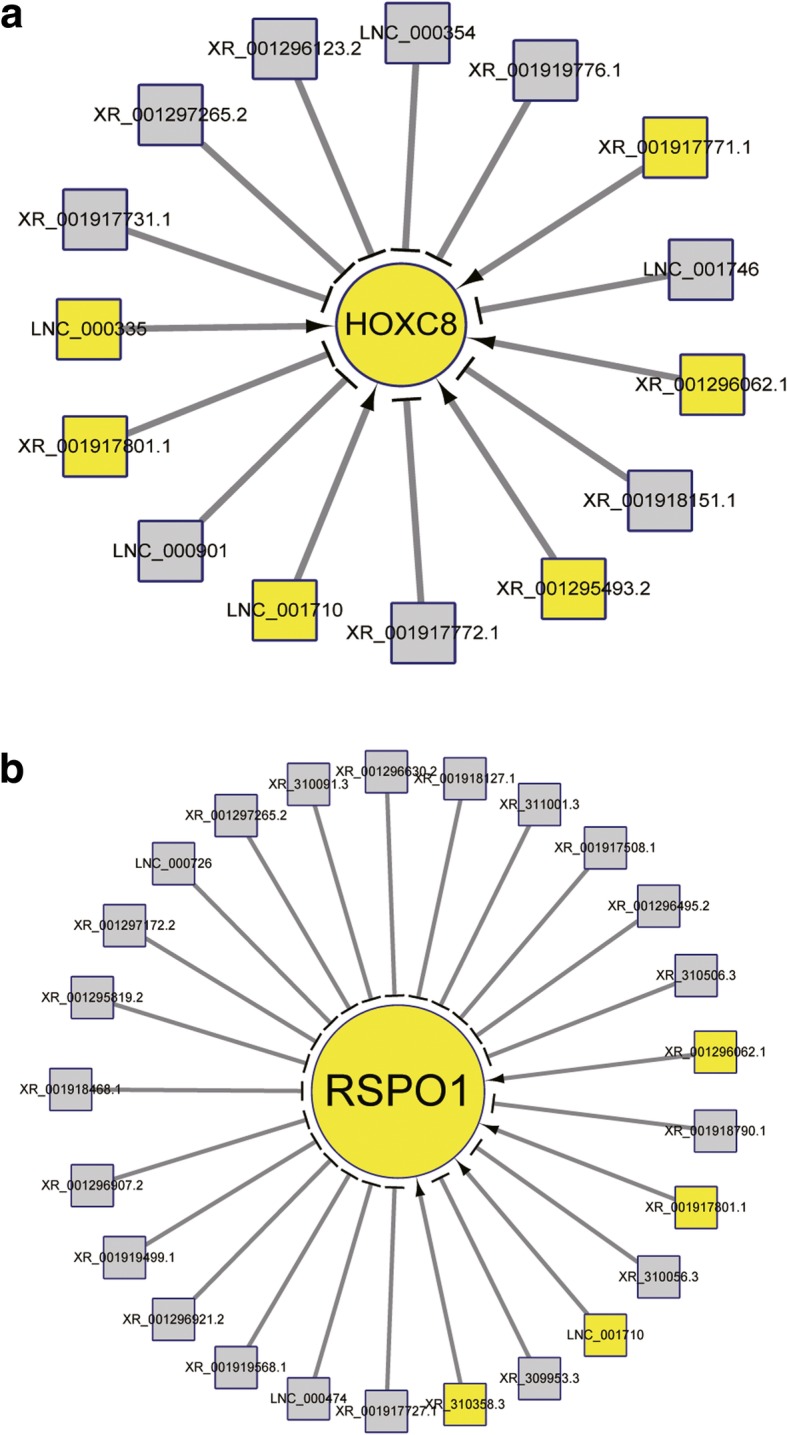
Potential lncRNAs targeting *HOXC8* (**a**) and *RSPO1* (**b**). Multiple lncRNAs target the two genes. *HOXC8* and *RSPO1* were indicated as circle with yellow color. LncRNAs were indicated as squares, yellow and grey color represents upregulated and downregulated lncRNAs in goat DPCs compared with DFs, respectively

MiRNAs exert their physiological roles via specifically binding, repressing translation and promoting degradation of their targeted mRNAs [61]. To infer the mighty functions of miRNAs in DPCs, we constructed their interactive network with core signatures (Fig. 3; Additional file 8: Table S4). As a result, we found that two HFSCs activation-related genes are regulated by several miRNAs. *HOXC8*, a crucial gene for HFSCs activation [60], is the target of chi-miR-144-5p. Another gene *RSPO1* with similar character in hair cycling is negatively modulated by chi-miR-874-3p, chi-miR-23b-5p and other five miRNAs. There are several ways for lncRNAs to exert their modulatory roles on gene expression, including as an enhancer, epigenetic modifier and transcriptional regulator [62]. Commonly, two modes *cis*-role (in which lncRNAs act on adjacent genes with 100 kb distances) and *trans*-role (in which the Pearson correlation of mutual expression levels is ≥0.95 or ≤ − 0.95) are widely adopted by researchers to forecast lncRNAs-gene interactive pair [2, 5]. Here, we focused on the possible relations of lncRNAs to HFSCs vitalization, and picked the two functional genes *HOXC8* [60] and *RSPO1* [63] as the targets. Finally, our results suggested that four lncRNAs fit the criterion of *cis*-role, but only LNC_000354, who situates 63,083 bp upstream of *HOXC8* locus, displayed differential expression (downregulated in DPCs), potentially imposing an inhibitive effect on *HOXC8*. For trans-acting pattern, we detected 13 lncRNAs, comprising 6 (e.g., XR_001296062.1 and LNC_001710) positively and 7 (e.g., XR_001919776.1 and XR_001918151.1) negatively correlated, respectively (Fig. 4a). Among these lncRNAs, some such as XR_001296062.1 are uniquely expressed in DPCs, whereas LNC_000901 possesses a DFs-specific expression. As for *RSPO1*, none lncRNAs act as *cis*-regulator and 26 lncRNAs work in *trans*-manner (Fig. 4b). Some regulatory lncRNAs also showed a specific expressing pattern in goat DPCs and DFs. Altogether, we implied that lncRNAs might affect HFSCs status via regulating gene expressions inside DPCs.

We also provide entire interactive relationships among core signatures and ncRNAs in Additional file 9: Figure S5.

### LncRNAs serve as ceRNAs to indirectly regulate *HOXC8* and *RSPO1*

**Fig. 5:**
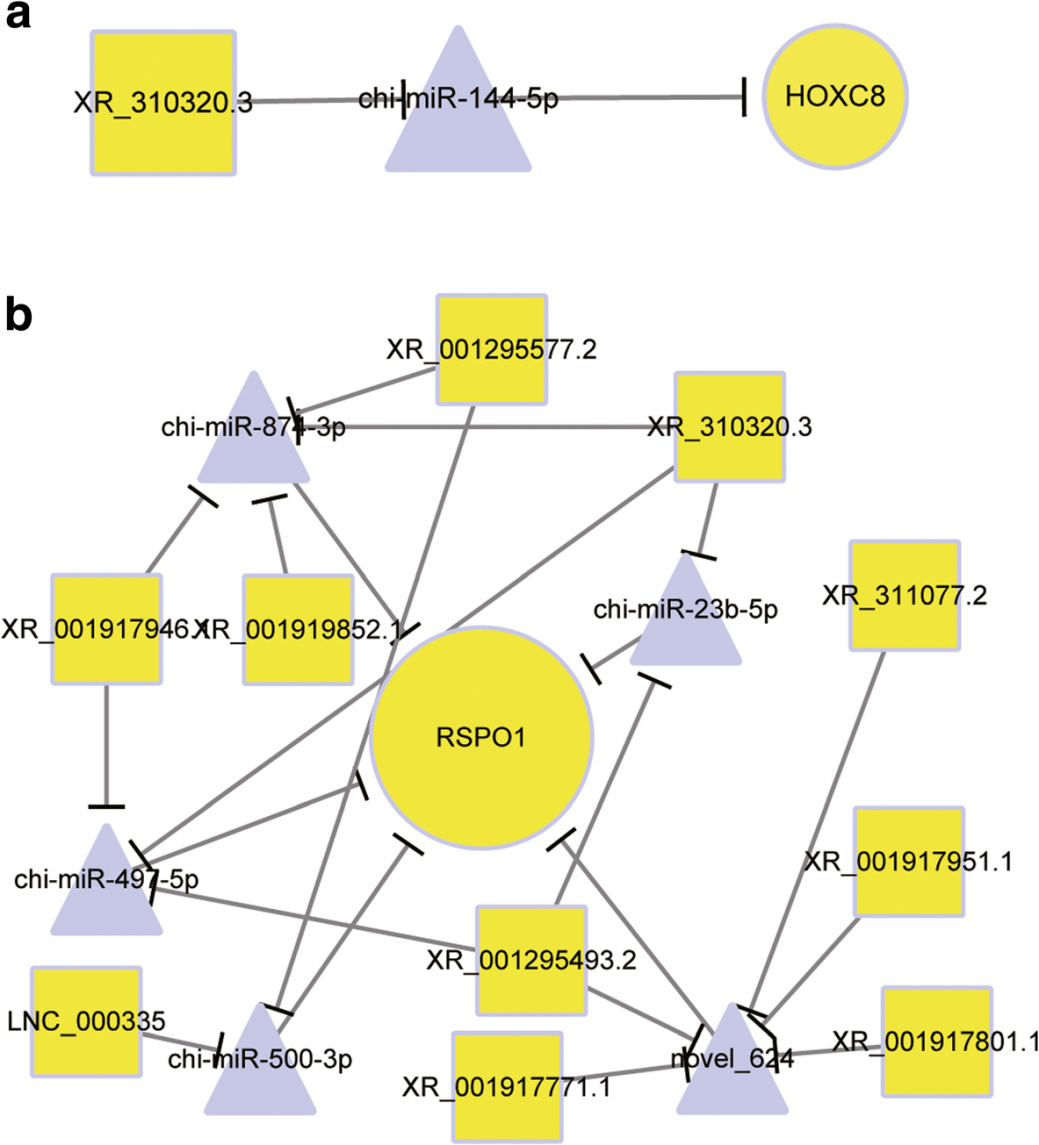
LncRNAs serve as ceRNAs. **a** XR_310320.3 functions as ceRNAs to upregulate *HOXC8*. **b** XR_001295577.2, XR_311077.2 and other eight lncRNAs function as ceRNAs to upregulate *RSPO1*. Circle represents mRNAs, square represents lncRNAs and triangle represents miRNAs. Yellow color indicates upregulated transcripts and blue color represent downregulated transcripts. LncRNAs were assumed to specifically sponge miRNAs to relief their suppressive roles on targeted mRNAs expression. The targeted relationship of miRNAs with mRNAs/lncRNAs were predicted using miRanda

Extensive studies discovered that lncRNAs harbor microRNA (miRNA)-response elements (MRE), and can serve as ceRNAs to decoy miRNAs, resulting in indirect upregulation of associated mRNAs [64]. Extensive pivotal roles concerning lncRNAs as ceRNAs have been reported on goats and other animals [21, 62, 65]. Here, we identified lncRNAs that might function as ceRNAs using a combination of bioinformatic tools, and focused their regulatory relationships with *HOXC8* and *RSPO1*. Our results suggested that some lncRNAs specifically bind to certain miRNAs targeting the genes to relief associated mRNAs from suppression, and thus modulate gene expressions as ceRNAs, such as XR_310320.3-chi-miR-144-5p-*HOXC8* and XR_311077.2-novel_624-RSPO1 interactive lines (Fig. 5). XR_310320.3 and XR_311077.2 act as ceRNAs to positively regulate *HOXC8* and *RSPO1* expressions*,* respectively*.* We showed the possibility that lncRNAs act as ceRNAs to participate in the regulation of HFSCs activation. Whole list of interactive pairs was provided in Additional file: Table S6.

## Discussion

Mammalian mature HF is a complicated miniorgan, mainly composing diverse types of epithelial cells. Although it is largely epithelium originated, HF contains a flock of specialized fibroblasts at its bottom, DPCs, who play pivotal roles in the regulation of continual regeneration of HF [66]. As hair regrowth initiates, signals secreted by DPCs are deemed to direct HFSCs locating in the bulge region of HF to proceed transit division. Then, stem cell progenies migrate downward into the base of HF, where they encircle the DPCs, forming the HMCs. Receiving further instructive signals from DPCs, HMCs serially undergo rapid proliferation and terminal differentiation to form keratinized hair shaft [7, 67]. Though unquestionable importance of DPCs in hair cycling, regulatory mechanisms regarding the expressions of such molecules inside DPCs are not well understood yet. In the present study, we executed a comprehensive ncRNAs and coding genes expression analysis between DPCs and DFs of cashmere goats, providing new views of ncRNAs in hair cycling.

We initially acquired DPCs and DFs from goat skins, and exhibited they possess obviously heterogeneous external appearances. This inconformity was identical with human [68], mice [36] and other animals [69, 70]. Previous studies found that skin implantation of cultured DPCs induced new hair growth, whereas DFs can’t [36, 71]. Above facts built a solid foundation for subsequent trial. Next, we carried out a comprehensive RNA-seq of both cell populations, and proceeded a series of bioinformatic analysis. Functional specificity of a cell is determined by its unique gene expression pattern, which is further controlled by diverse factors [56, 72]. Several reports on humans and mice have unveiled the crucial characters of ncRNAs in modulating the functionality of DPCs during hair growth. Indeed, altered profiles of miRNAs have been frequently connected to dysfunctions of human and mice DPCs, and recently some lncRNAs were also found as casual elements of such conditions [28]. However, previous studies mainly focused on humans and mice, little information was known about the functions of ncRNAs in goats, especially for less conservative lncRNAs [62]. In present study, we discovered that a plenty of miRNAs and lncRNAs were abundantly present in DPCs and DFs, and displayed remarkably differential expressions between two cell types. Unique expressions of certain miRNAs (e.g., novel_144 and chi-let-7c-3p) and lncRNAs (e.g., LNC_001710 and LNC_000799) in individual cell lineage were also discerned (Table 2), suggesting they might participate in the regulation of cellular functions in a cell type-restricted manner [15]. Our further analysis showed that a sum of 86 miRNAs, 71 lncRNAs and 2538 mRNAs transcripts were differentially expressed between DPCs and DFs. Some miRNAs (e.g., chi-miR-1271-5p, chi-miR-9-5p, and chi-miR-22-3p) and lncRNAs (e.g., LNC_000349, XR_001297172.2, and XR_310056.3) were previously identified as differentially expressed transcripts among stage transitions of cashmere growth [2, 3, 5]. However, additional cautions should be needed because these studies profiled ncRNAs from skin tissue, a complex structure comprising diverse kinds of cell including epidermal keratinocytes, fibroblasts, intradermal adipocytes, as well as HF itself. The majority of them experience drastic fluctuation in morphologies and gene expressions during hair cycling [7]. We also found that two miRNAs miR-125b (downregulated in DPCs) and miR-335 (upregulated in DPCs) deserve more attention. miR-125b was shown to decrease level of *FGF7*, a potent growth factor secreted by goat DPCs [27]. miR-335 was positively correlated with the hair-inducing capacity of mice DPCs [26]. Despite the truth that the functions of ncRNAs in DPCs have not been extensively explored yet, their remarkably distinct expression patterns between DPCs and DFs suggest they should be a pivotal participator of hair growth regulation. Compared with previous studies on complex skin tissue of goats, the present single cell type transcriptomes could offer reliable and valuable candidates for downstream functional verification. On the other hand, synchronous detection of ncRNAs and mRNAs enables us to faithfully construct relevant interplay network among diverse transcripts and further deduce unearthed mechanisms of ncRNAs, which have been pervasively done in other cells or tissues [30, 32, 73], however sparsely reported in mammalian DPCs.

The HF is extraordinarily sensitive to locally and systematically generated hormones that modulate hair growth, leading to an adaptive accommodation to external environment, such as climate change [74]. Hormone-participated regulation makes sure that the HF timely transits among different developmental stages [75]. We found that three hormone-related pathways Estrogen signaling pathway, Thyroid hormone signaling and Adipocytokine signaling pathway were enriched in our KEGG analysis and corresponding hormone receptors (i.e., *ESR1*, *IGTAV* and *LEPR*) were significantly upregulated in DPCs compared with DFs. These pathways were also frequently appeared in recent reports on cashmere periodical development [2, 3, 5], implying DPCs are the follicular targets of these hormones. These results are also in consistent with the discoveries on humans and mice [57, 76]. Leptin and thyroid hormone markedly prolonged growing phase of HF [77, 78], whereas estrogen exerted an inhibitive effect on hair growth [79]. Though crucial roles of these hormones on hair development, working approaches in DPCs are not clear yet. A series of intraceullar kinases and transcription factors are responsible for corresponding signaling transductions and gene expression modulations of the hormones [80, 81]. Some of them (e.g., *JAK2*, *MAPK1*, *PIK3R1*, *JUN1* and *SP1*) were highly expressed in goat DPCs, thus providing useful clues for further researches.

We further defined 25 core signatures of DPCs through overlapping present result with a previously published data of mice [56]. Among these genes, the functions of the majority of them were well-characterized on mice models. Induced activation of HFSCs by signals emanating from DPCs drives HF reconstruction and hair regrowth [82]. Here, we found that *HOXC8*, *RSPO1* and *BMP4* are the involved molecules. *RSPO1,* a secreted Wnt/β-cateinin agonist, is highly enriched in mice DPCs, and its recombinant protein injection into mice skins resulted in vitalizing HFSCs and precocious anagen entry from mid-telogen [63]. The transcription factor, *HOXC8,* exerted similar influences on hair cycling through acting as an upstream positive regulator of Wnt signaling [60]. Of greatly noticeable, the hypermethylation status of *HOXC8* exon1 is associated with shorter fleece length on cashmere goats [6], and first exon methylation tightly concerns with transcriptional silencing [83]. These observations demonstrate that artificial alteration of HFSCs status through manipulating gene expression of DPCs is a hopeful strategy for enhancing fiber productions in farm animals. Another gene, BMP4 was reportedly suppressing HFSCs in quiescent state [84]. Interestingly, melatonin, the famous hormone affects seasonal growth of cashmere, seems to intimately interplay with both Wnt/β-cateinin and *BMP4* in other tissues and systems [85]. Whether it acts in the same manner in DPCs should be determined in future.

Accumulating evidences suggest miRNAs and lncRNAs impose an indispensable modulatory role on gene expression [21]. Compared with a sole approach of miRNAs, lncRNAs can perform as enhancers, transcriptional regulators, miRNAs sponges and others, which are generally categorized as *cis*- and *trans*- regulators [16]. Several paradigms have been well appreciated such as Xist acting as a cis-modulator for genomic imprinting and HOTAIR serving as trans-regulator for HOXD gene silencing [19, 86]. Past reports have identified a few ncRNAs and uncovered their vital functions in DPCs, such as miR-125b, miR-195p, HOTAIR and LncRNA-000133 [26–29]. Whereas, there are still no reports focused on the connections of ncRNAs with DPCs-mediated HFSCs activation. In this study, we established the regulatory relationships of miRNAs and lncRNAs with *HOXC8* and *RSPO1.* Our results suggested that some miRNA and lncRNAs can specifically target the genes and impose negative or positive effects of their expressions, implying the possible characters of ncRNAs in HFSCs vitalization. Otherwise, the crucial characters of lncRNAs functioning as ceRNAs in stem cell biology have also been demonstrated, such as linc-RoR specifically decoys miR-145, upregulates Oct4, Nanog and Sox2 and contributes to the self-renewal of embryonic stem cells [87]. We also identified lncRNAs might serve as ceRNAs to sponge miRNAs and modulate the levels of two genes, and built related interactive lines such as XR_310320.3-chi-miR-144-5p-*HOXC8* and XR_311077.2-novel_624-*RSPO1*. XR_310320.3 and XR_311077.2 may work as potent ceRNAs to particularly enhance the expressions of *HOXC8* and *RSPO1*, resulting indirect vitalization of HFSCs. Overall, we exhibited multiple facets of lncRNAs in DPCs, and their critical importance in adjusting HFSCs status. These will greatly facilitate the understanding of ncRNAs in DPCs and HF development.

## Conclusion

In present study, we reported the expression profiles of miRNAs and lncRNAs, as well as mRNAs in goat DPCs and DFs. Through definition of core signatures of DPCs and construction of their regulatory network with ncRNAs, we exhibited the specific modulation of ncRNAs on *HOXC8* and *RSPO1*, the genes involved in HFSCs activation. Our study provided specific transcriptomic data for cashmere goat research and hinted the potential roles of ncRNAs in HF development and cycling.

## Methods

### Cell cultivation

The Shanbei cashmere goats were carefully managed in a farm located in the Yangling District, Shaanxi Province, China. We selected three healthy 2-year-old female Shanbei White Cashmere goats with similar body weights (~ 35 kg) and uncrossed lineage records for the current study. Subcutaneous procaine injection was performed to alleviate animal suffering. In September (anagen phase of cashmere growth), approximately 2 cm^2^ skin samples on lateral backsides of the goats were surgically removed under standard anesthetic and aseptic procedures. After suturing all wounds, we intentionally took good care of experimental goats to boost their recovery from surgery. The excised skin samples were washed three times with sterile phosphate-buffered solution (PBS) to clean off blood or other contaminants. Then, the tissues were cut at the interface of the subcutaneous adipose layer and dermis. An incised subcutaneous adipose layer was applied for isolation of intact HF and in vitro cultivation of dermal papilla cells, as previously stated [39]. The remaining tissues were submerged in 0.25% dispase II overnight at 4 °C to separate the epidermis and dermis, then the dermis was cut into small blocks approximately 1 mm^3^ in size and used as explants for DFs culture. Normally, DPCs and DFs grew from each explant about five days after initial adhesion and were passaged when they reached 100% confluency. Typically, DPCs and DFs from the 4th passages were used for all of the experiments. Primary and passaging cultures of each cell type were maintained in DMEM/F12 supplemented with 10% FBS, 100 units/ml penicillin, and 100 μg/ml streptomycin, and the cultures were incubated at 37 °C and 100% humidity in a 5% CO_2_/ 95% air incubator.

### Total RNA extraction, library preparation, and sequencing

Six strains of cells (three for DPCs or DFs) without cross contamination were selected, and their total RNAs were extracted using the RNA isolation kit mentioned above according to the manufacturer’s instructions. To ensure the fidelity of co-expression analysis, two independent libraries were simultaneously constructed for each strain: the lncRNAs library (for mRNAs and lncRNAs) and the miRNAs library. The preparation of both libraries was performed as previously reported [2], and the resulting libraries were further sequenced on Illumina HiSeq 4000 (for mRNAs and lncRNAs) or HiSeq 2000 platforms (for miRNAs), which generated 150-bp paired-end and 50-bp single-end reads, respectively.

### Sequencing data analysis

To determine the landscape of transcript expression in all samples, clean reads were acquired by removing reads containing poly-N, along with low-quality and other invalid reads among all of raw reads. At the same time, calculations of the Phred score (Q20, Q30) and GC content of the clean data were also performed before downstream analysis. For lncRNAs library mapping, Bowtie2 (v2.2.8) was employed to assess the index of the reference genome and paired-end clean reads were aligned to the reference genome using HISAT2 (v2.0.4) [42]. At the same time, Bowtie (v0.12.9) was used to map small RNA tags to the reference sequence without mismatch to analyze their expression and distribution on the reference. Assembly of mapped reads was achieved using StringTie (v1.3.1) in a reference-based approach. Cuffdiff (v2.1.1) was used to calculate the fragments per kilo-base of exons per million fragments mapped (FPKMs), and the Ballgown suite was adopted for the determination of differentially expressed lncRNAs and mRNAs transcripts [42]. LncRNAs were distinguished based on several key well-known criteria: exon number (> 2); transcripts length (> 200 nt); abundance (FPKM > 0.5); and protein-coding potential, which were excluded by the CNCI (v2) [46], CPC (cpc-0.9-r2) [44] and Pfam-scan (v1.3) [45] tools. For target gene prediction of lncRNAs, the *cis*-role was set as lncRNAs act on neighboring target genes, which were defined as genes located 100 k upstream and downstream of lncRNAs locus. Expression correlations between lncRNAs and coding genes were calculated to predict the *trans*-role of lncRNAs.

MiRBase20.0 was chosen as the reference to filter known miRNAs, and novel miRNAs were predicted using miREvo [47] and mirdeep2 [48] through the exploration of the secondary structure, the dicer cleavage sites and others. Prediction of the target gene of miRNAs was carried out by miRanda [88], and miRNA expression abundances were estimated by transcript per million (TPM) using the following criteria: normalized expression = mapped readcount/total reads*1000000. Differential expression of miRNAs among samples was analyzed using the DESeq R package (1.8.3) [89].

### cDNA synthesis and quantitative real-time PCR (qPCR)

Total RNA extracted from cell samples was used for qPCR validation of sequencing data or other data. First strand cDNA was synthesized using the cDNA Synthesis Kit according to the manufacturer’s instructions and then was subjected to qPCR experiments on a Bio-Rad CFX96 Real-Time PCR Detection System. qPCR assays were executed as the manufacturer suggested. Detailed sequences of primers used for this study are listed in Additional file 10: Table S6, and *GAPDH* was used to normalize gene expression. Three biological and technical replicates were set for all experiments. The 2^-ΔΔCt^ method was employed to calculate relative transcript expression.

### KEGG pathway analysis

As reported in previous studies [90, 91], we used the KOBAS3.0 [92] program to arrange the differentially expressed transcripts into their involved KEGG pathways.

### Construction of interactive networks among differentially expressed transcripts

To better understand the regulatory relationships of noncoding genes with mRNAs, and further reveal their functions in hair biology, we constructed interaction networks among these transcripts. As many researchers did before [2, 51], the predictions of the target transcripts of miRNAs, including lncRNAs and mRNAs, were achieved using miRanda [88], which is a program exclusively developed for animals. Based on co-location and co-expression, we constructed an interacting network of mRNAs-lncRNAs. Likewise, based on the prediction of miRNA binding sites inside mRNAs or lncRNAs, and their expression level relevance, we built networks of mRNAs-miRNAs and lncRNAs-miRNAs. The other types of interacting pairs were created with the popular ceRNAs hypothesis in which lncRNAs act as sponges to absorb miRNAs and then free its suppressive roles on mRNA transcription or translation. Finally, lncRNAs-miRNAs-mRNAs interactive lines were fabricated. All results were graphically displayed using Cytoscape3.6.

## Additional files

Additional file 1: **Figure S1.** Identification of lncRNAs in goat DPCs and DFs. **a** Five successive screening steps of valid lncRNAs transcripts from all samples: step 1: exon counts (> 2); step 2: transcript length (> 200 nt); step 3: expression abundance (FPKM > 0.5); step 4: preclusion of known lncRNAs transcripts in the database and step 5: the absence of protein-coding potential. **b** Coding potential prediction of lncRNAs candidates using CPC, CNCI and PFAM tools. Overall, 1754 lncRNAs transcripts that are not supposed to code protein according to any tool were thought to be novel lncRNAs (overlapped region) and proceeded to downstream analysis. (TIF 6882 kb)

Additional file 2: **Table S1.** Information of lncRNAs transcripts. (XLSX 209 kb)

Additional file 3: **Figure S3.** Comparison of the genomic features of lncRNAs and mRNAs. Density distribution of exon counts (**a**) and transcript length (**b**) in annotated lncRNAs, novel lncRNAs and mRNAs. Usually, mRNAs contain more exons and nucleotides than lncRNAs. **c** Box plot showing the abundances of transcripts, and the expression levels of mRNAs are higher than annotated lncRNAs or novel lncRNAs as indicated. (TIF 8461 kb)

Additional file 4: **Figure S4.** Heatmaps of differentially expressed transcripts. **a** Heatmap of differentially mRNAs. **b** Heatmap of differentially lncRNAs. **c** Heatmap of differentially miRNAs. Compared with DFs, 1286 mRNAs, 18 lncRNAs, and 42 miRNAs were upregulated, while 1254 mRNAs, 53 lncRNAs and 44 miRNAs were downregulated in DPCs. (TIF 22373 kb)

Additional file 5: **Table S2.** Lists of differentially expressed transcripts. (XLSX 261 kb)

Additional file 6: **Figure S5.** Validation of relative abundances of transcripts in DPCs and DFs by qPCR. *GAPDH* was adopted as the internal reference to normalize all gene expression. Relative gene expressions were acquired through the classic comparative cycle threshold method. Fold change was calculated as the mean of three independent samples. Similar trends were found in all examined genes. (TIF 3019 kb)

Additional file 7: **Table S3.** Enriched pathways of upregulated genes in DPCs. (XLS 144 kb)

Additional file 8: **Table S4.** Interactive relationship of ncRNAs with core signatures. (XLSX 60 kb)

Additional file 9: **Table S5.** Interactive lines of ncRNAs with core signatures. (XLSX 38 kb)

Additional file 10: **Table S6.** Primer information used in present study. (XLSX 9 kb)

## Data Availability

The transcriptomic data generated for this study were deposited in the NCBI Sequence Read Archive (http://www.ncbi.nlm.nih.gov/sra). All accession numbers are listed as follows: SRR8138850, SRR8138851 and SRR8138852 for lncRNAs of DPCs; SRR8138849, SRR8138855 and SRR8138856 for lncRNAs of DFs; SRR8138853, SRR8138854 and SRR8138858 for miRNAs of DPCs; SRR8138857, SRR8138859 and SRR8138860 for miRNAs of DFs.

## Abbreviations

*BMP4*: Bone morphogenetic protein 4
*COL15A1*: Collagen type XV alpha 1 chain
*CRABP1*: Cellular retinoic acid-binding protein 1
DFs: Dermal fibroblasts
DPCs: Dermal papilla cells
*ESR1*: Estrogen receptor alpha
FGF: Fibroblast growth factor
*FGFR2*: Fibroblast growth factor receptor 2
FPKM: Fragments per kilobase of transcript per million mapped reads
HF: Hair follicle
HFSCs: Hair follicle stem cell
HMCs: Hair matrix cells
*HOTAIR*: Transcript antisense RNA
*HOX*: Homeobox protein
*ITGA*: Integrin alpha
*LEPR*: Leptin receptor
lncRNAs: Long non-coding RNAs
*LTBP1*: Latent-transforming growth factor beta-binding
ncRNAs: Non-coding RNAs
*PRDM1*: PR domain zinc finger protein 1
*PTGER4*: Prostaglandin EP4 receptor
*PTGFR*: Prostaglandin F receptor
*RSPO1*: R-spondin 1
*SOX2*: SRY (sex determining region Y)-box 2
*TMEM100*: Transmembrane protein 100
*WNT5A*: Wnt Family Member 5A
