# Supplementary figures and images for: Synchronous profiling and analysis of mRNAs and ncRNAs in the dermal papilla cells from cashmere goats
Source: BMC Genomics. 2019 Jun 20;20:512. doi: 10.1186/s12864-019-5861-4 (PMC6587304; doi:10.1186/s12864-019-5861-4)

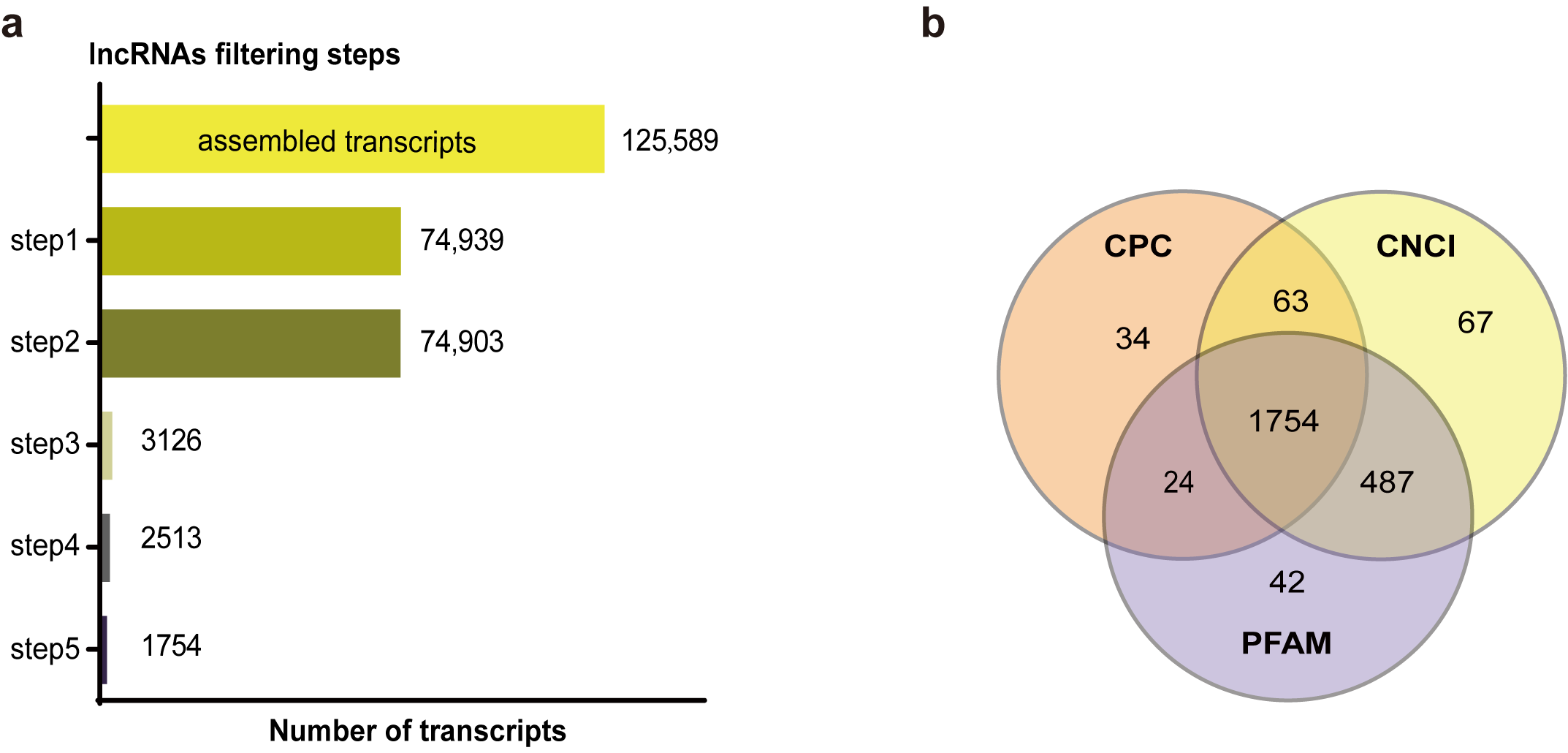

Supplement: Supplementary file 1 — Figure S1. Identification of lncRNAs in goat DPCs and DFs. a Five successive screening steps of valid lncRNAs transcripts from all samples: step 1: exon counts (> 2); step 2: transcript length (> 200 nt); step 3: expression abundance (FPKM > 0.5); step 4: preclusion of known lncRNAs transcripts in the database and step 5: the absence of protein-coding potential. b Coding potential prediction of lncRNAs candidates using CPC, CNCI and PFAM tools. Overall, 1754 lncRNAs transcripts that are not supposed to code protein according to any tool were thought to be novel lncRNAs (overlapped region) and proceeded to downstream analysis. (TIF 6882 kb) [file 12864_2019_5861_MOESM1_ESM.tif]

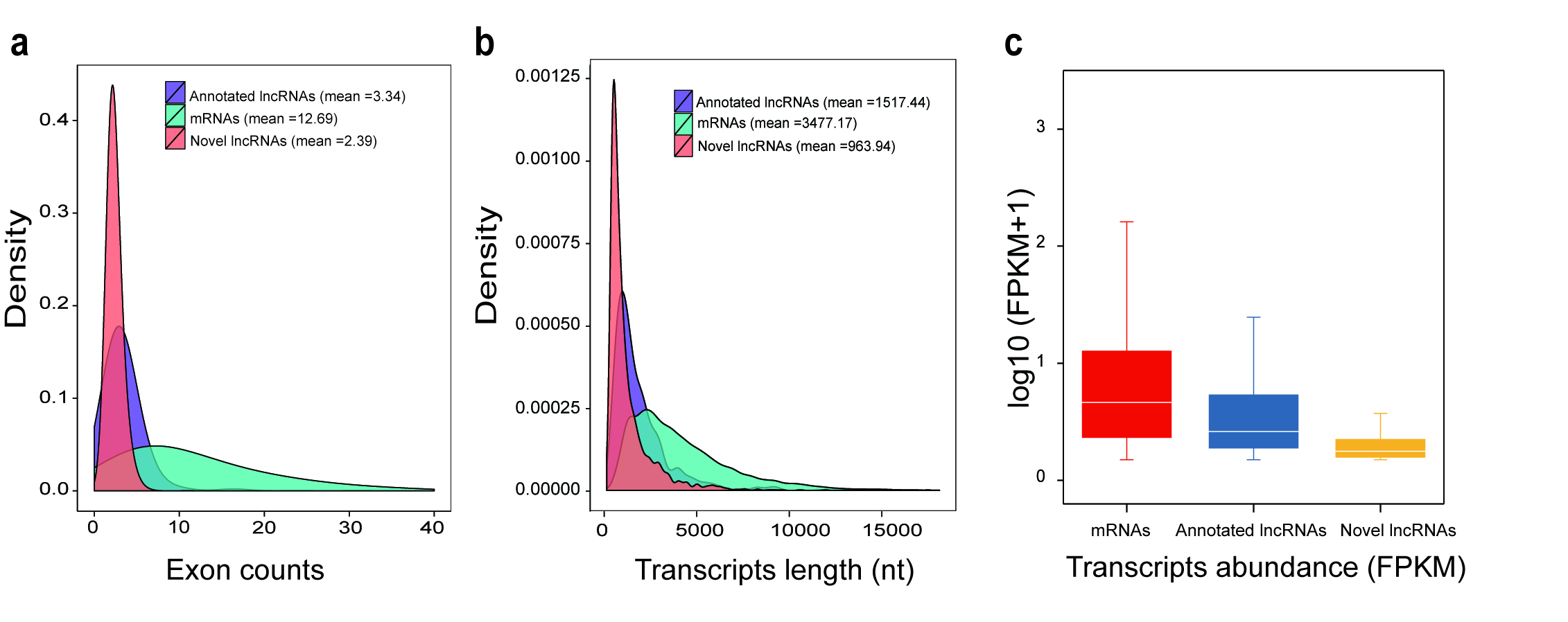

Supplement: Supplementary file 3 — Figure S3. Comparison of the genomic features of lncRNAs and mRNAs. Density distribution of exon counts (a) and transcript length (b) in annotated lncRNAs, novel lncRNAs and mRNAs. Usually, mRNAs contain more exons and nucleotides than lncRNAs. c Box plot showing the abundances of transcripts, and the expression levels of mRNAs are higher than annotated lncRNAs or novel lncRNAs as indicated. (TIF 8461 kb) [file 12864_2019_5861_MOESM3_ESM.tif]

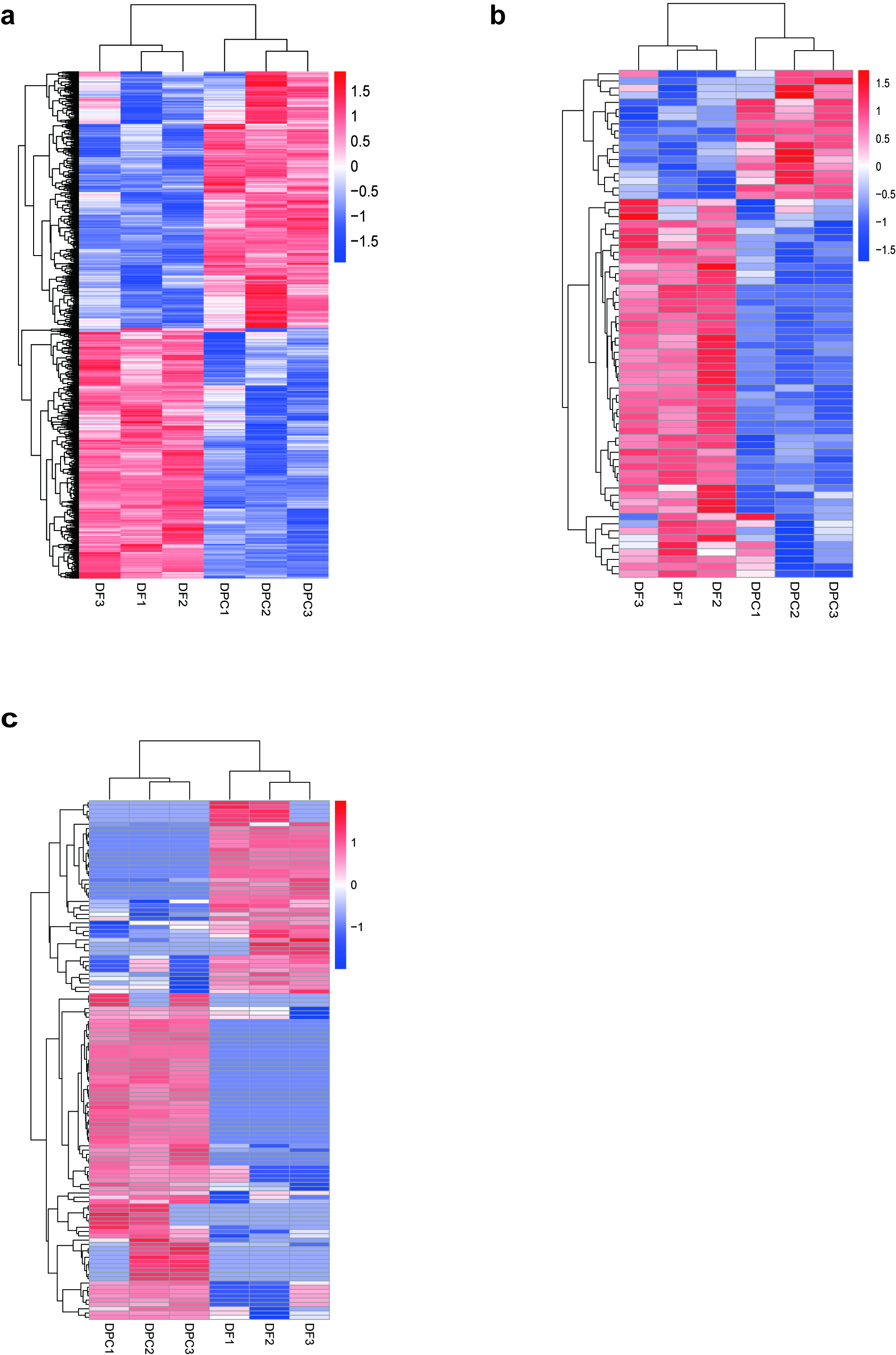

Supplement: Supplementary file 4 — Figure S4. Heatmaps of differentially expressed transcripts. a Heatmap of differentially mRNAs. b Heatmap of differentially lncRNAs. c Heatmap of differentially miRNAs. Compared with DFs, 1286 mRNAs, 18 lncRNAs, and 42 miRNAs were upregulated, while 1254 mRNAs, 53 lncRNAs and 44 miRNAs were downregulated in DPCs. (TIF 22373 kb) [file 12864_2019_5861_MOESM4_ESM.tif]

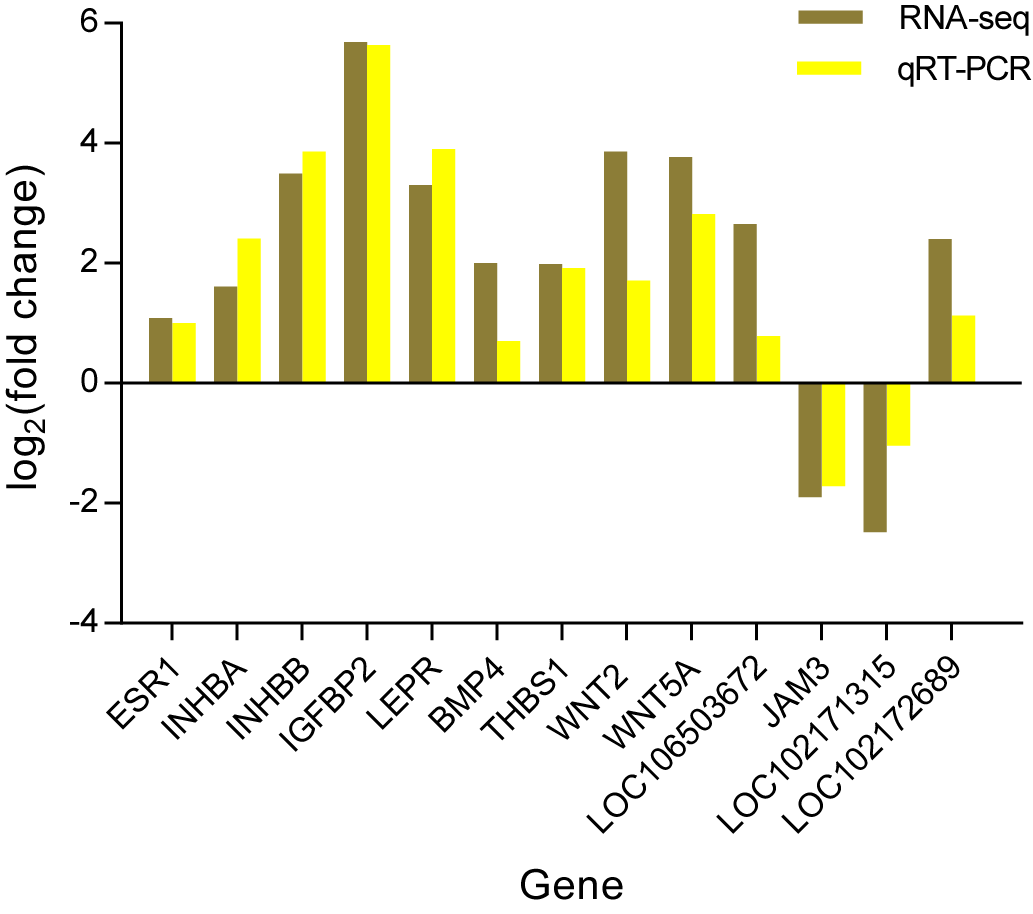

Supplement: Supplementary file 6 — Figure S5. Validation of relative abundances of transcripts in DPCs and DFs by qPCR. GAPDH was adopted as the internal reference to normalize all gene expression. Relative gene expressions were acquired through the classic comparative cycle threshold method. Fold change was calculated as the mean of three independent samples. Similar trends were found in all examined genes. (TIF 3019 kb) [file 12864_2019_5861_MOESM6_ESM.tif]
